# The Role of Platelets in the Pathogenesis and Pathophysiology of Adenomyosis

**DOI:** 10.3390/jcm12030842

**Published:** 2023-01-20

**Authors:** Sun-Wei Guo

**Affiliations:** 1 Shanghai Obstetrics and Gynecology Hospital, Fudan University, Shanghai 200011, China; hoxa10@outlook.com; Tel.: +86-21-3318-9900 (ext. 326); Fax: +86-21-5302-8000; 2 Shanghai Key Laboratory of Female Reproductive Endocrine-Related Diseases, Fudan University, Shanghai 200011, China

**Keywords:** adenomyosis, coagulation, dysmenorrhea, fibrogenesis, heavy menstrual bleeding, repeated tissue injury and repair

## Abstract

Widely viewed as an enigmatic disease, adenomyosis is a common gynecological disease with bewildering pathogenesis and pathophysiology. One defining hallmark of adenomyotic lesions is cyclic bleeding as in eutopic endometrium, yet bleeding is a quintessential trademark of tissue injury, which is invariably followed by tissue repair. Consequently, adenomyotic lesions resemble wounds. Following each bleeding episode, adenomyotic lesions undergo tissue repair, and, as such, platelets are the first responder that heralds the subsequent tissue repair. This repeated tissue injury and repair (ReTIAR) would elicit several key molecular events crucial for lesional progression, eventually leading to lesional fibrosis. Platelets interact with adenomyotic cells and actively participate in these events, promoting the lesional progression and fibrogenesis. Lesional fibrosis may also be propagated into their neighboring endometrial–myometrial interface and then to eutopic endometrium, impairing endometrial repair and causing heavy menstrual bleeding. Moreover, lesional progression may result in hyperinnervation and an enlarged uterus. In this review, the role of platelets in the pathogenesis, progression, and pathophysiology is reviewed, along with the therapeutic implication. In addition, I shall demonstrate how the notion of ReTIAR provides a much needed framework to tether to and piece together many seemingly unrelated findings and how it helps to make useful predictions.

## 1. Introduction

Adenomyosis, defined as the presence of endometrial glands and stroma within the myometrium [1], is a uterine disease that affects many women of reproductive age and contributes to dysmenorrhea, pelvic pain, abnormal uterine bleeding (AUB)/heavy menstrual bleeding (HMB), and subfertility [2,3,4,5]. While approximately one third of women with adenomyosis are asymptomatic [6], dysmenorrhea is the most prevalent symptom besides HMB [7]. As a result, adenomyosis negatively impacts the quality of life of the afflicted woman [8]. It is also associated with an increased risk of several adverse pregnancy outcomes [3,9].

Notably, adenomyosis shares many similarities with endometriosis. Both diseases are estrogen-dependent [10], have similar symptomology, and share many molecular aberrations, such as an increased production of proinflammatory cytokines/chemokines [11,12], overexpression of COX-2 [13], increased oxidative stress [14,15,16,17] and angiogenesis [18], epigenetic aberration [19,20], and cancer-driver mutations [21]. Not surprisingly, the management strategies for the two diseases are also similar, and the drugs for treating endometriosis are also used for treating adenomyosis [22]. In fact, few drugs are being developed exclusively for adenomyosis [23].

Yet perhaps the most glaring feature shared by both diseases is cyclic bleeding [24], so much so that one salient commonality conspicuously shared by all current hormonal drugs for treating adenomyosis/endometriosis is that they all arrest cyclic bleeding [24]. However, bleeding is a hallmark of vascular injury and thus tissue injury. In all organisms, following a tissue injury, the evolutionarily conserved tissue repair program will immediately kick in. In other words, adenomyotic lesions thus resemble wounds that undergo repeated tissue injury and repair (ReTIAR) [25]. Consequently, they would experience the well-known four phases in tissue repair: hemostasis, inflammation, proliferation, and remodeling. Among all these phases, platelets are the first to rush to the wounded site, heralding the repair process [26]. Thus, it is quite fitting to review the roles of platelets in adenomyosis, especially in its progression. In addition, since HMB is known to result from impaired endometrial repair [27], it is conceivable that platelets may also have a role in adenomyosis-induced HMB, although research in this area has been surprisingly scanty so far.

Moreover, there are numerous case reports documenting thromboembolism, cerebral infarction, and disseminated intravascular coagulation in women with adenomyosis [28,29,30,31,32,33]. This raises the prospect that women with adenomyosis, especially those with HMB or severe pain, may be hypercoagulable, which, again, hints at the role of platelets.

There have been 3449 papers on adenomyosis as indexed by PubMed (accessed on 14 November 2022), and well over half of them (*n* = 1941 or 56.3%) were published in the last decade. Many of these publications helped us better understand the pathogenesis and pathophysiology of adenomyosis. We often see very clearly the leaves, twigs, branches, or even a single tree, but unfortunately, we seldom see the forest or the bigger picture. Why is there such an aberration? What is the natural history of the adenomyotic lesion? Is there any theme or framework that can be used to tether to and piece together most, if not all, published findings? Can we make useful predictions to guide our future research?

Without the ReTIAR theme, however, it is difficult to think of any framework that can thether to and piece together many published but seemingly isolated findings. The entire field is like what was depicted in the famous fable on the elephant and the blind men; each of these blind men told a certain truth, but certainly not the whole and the entire truth. However, if we see through the ReTIAR prism, we would realize that the prism is not only useful to assist us in tethering to and piecing together many seemingly unrelated findings, but, more importantly, can also help us to foresee things that we are otherwise completely unaware of.

Within this framework, platelets can be viewed as an important player in adenomyosis. In this review, I shall first provide a brief overview on tissue repair, platelets, and coagulation, and then I will provide a narrative review on the roles of platelets in the pathogenesis and pathophysiology of adenomyosis, piecing together some scattered and seemingly related findings and also attempting to make some useful predictions for future research.

## 2. A Primer on Tissue Repair, Platelets, and the Coagulation Pathways

Tissue repair, especially dermal tissue repair, has been extensively investigated [34,35]. Following tissue injury, tissue repair or wound healing ensues, which undergoes four distinct but somewhat overlapping phases: hemostasis, inflammation, proliferation, and resolution or remodeling [35]. Upon tissue injury, platelets, as the first-aid cells, are activated and aggregated into the site of the injury, resulting in the formation of a fibrin clot consisting of a network of insoluble fibrin fibers. Besides plugging vessels to stop the hemorrhage, the clot also acts as a provisional matrix to which growth factors bind and through which cells can crawl [36]. The damaged cells also release endogenous molecules, including damage-associated molecular pattern molecules (DAMPs), which may activate pattern recognition receptors (PRRs) and act as activation cues and/or chemotactic factors for other cells in the area [34]. Cells bathed in the serum—the fluid component of clotted blood—which contains many interleukins (ILs), colony-stimulating factors, TNF-α, IFN-γ, and other components, would activate the serum response factor (SRF) that binds and induces transcription of immediate early and other genes, such as c-Fos and early growth response genes [34]. DAMPs, H_2_O_2_, Ca^2+^, chemokines, and other mediators released by injured cells would recruit various inflammatory cells, including neutrophils and macrophages, ushering into the inflammatory phase. Neutrophils also produce neutrophil extracellular traps (NETs) that capture pathogens through a process called NETosis. Early macrophages in the wound, on the other hand, release MCP-1 to recruit more monocytes from the bone marrow and intensify the macrophage response. Macrophages also release pro-inflammatory cytokines, such as IL-6, TNF-α, and IL-1β, to combat infection and carry out phagocytosis of pathogens and tissue debris in the wounding site. At the end of the inflammatory phase, macrophages devour apoptotic neutrophils, ending the inflammatory phase of tissue repair and starting the proliferation phase. They also make transitions into an anti-inflammatory phenotype known as the alternatively activated macrophage or the M2 macrophage [35].

During the proliferation stage of tissue repair, macrophages release growth factors, such as VEGF and PDGF, to induce angiogenesis, which involves the activation of local microvascular endothelial cells. The final stage of tissue repair (i.e., remodeling) consists of the regression of the neovasculature, as well as apoptosis of myofibroblasts. Macrophages also play a phagocytic role where they devour both cell debris and excessive extracellular matrix (ECM), in order to bring the healed tissue to a homeostatic state.

Platelets are anucleated cells originating from cytoplasmic fragmentation of megakaryocytes primarily in the bone marrow. Packaged into several different containers called granules (α-granules, dense granules, and lysosome granules) with a plethora of pre-synthesized bioactive molecules, circulating platelets patrol in the blood of mammals and are critical effectors of hemostasis, blood clotting, and tissue repair [37]. Upon activation following exposure to damaged blood vessels, the surface receptors of platelets undergo a conformational change and bind to exposed sub-endothelial matrix proteins and von Willebrand Factor (vWF), resulting in morphological changes. The conformational change enables ligand binding and subsequent intracellular signaling cascades. The activated platelets create pseudopods, attaching to each other and releasing the contents of granules to initiate the aggregation process. They also bind to fibrinogen, resulting in the platelet plug that can effectively stop the hemorrhage from small injuries. They release ADP from their dense granules and also produce thromboxane A2 (TXA_2_) from arachidonic acid derived from phospholipids on their membrane. ADP and TXA_2_ further induce platelet activation [38].

Platelets play a pivotal role in the tissue injury/damage and inflammatory response, destined to initiate the repair of injured tissues. Erratic or uncontrolled platelet activation results in chronic inflammation associated with numeric pathological conditions, including cancer [39], fibrosis [40], and atherothrombosis [41]. Remarkably, platelets have an intricate transcriptome (with an entire repertoire of RNAs produced), activation-dependent post-transcriptional pathways, the capability of influencing extravascular events, and longer life spans than previously appreciated [42].

However, platelets are not merely the cells that mediate hemostasis. Over the years there has been growing evidence that these cells may act as key regulators in immune responses and are involved in the pathogenesis of various immune-mediated diseases, such as irritable bowel disease [43]. They can also function as antigen presenting cells and activate T cells through MHC-I [44]. In addition, platelets from patients with myocardial infarction can activate CD4+/CD69+ T cells and increase the number of regulatory T cells [45].

Upon tissue injury, the initial hemostatic response is triggered by tissue factor (TF) expressed on sub-endothelial pericytes and fibroblasts. Activated Factor VII (fVIIa) then binds to TF to activate fX to fXa, which generates trace amounts of thrombin [46]. There are two major inhibitors that regulate TF-triggered procoagulant responses, thus limiting serine protease actions to the site of vascular injury. TF pathway inhibitor (TFPI) neutralizes fXa when it is in a complex with TF-fVIIa. The other regulator of TF-trigger procoagulant response is antithrombin (AT, also known as AT III), which circulates at a high concentration and neutralizes the initially formed fXa and thrombin. Consequently, the procoagulant triggering reaction only proceeds when TF is exposed at a high enough level to overcome inhibition by TFPI and AT. That is, fVIIa circulates in the blood in search for sites of vascular damage (i.e., where TF is exposed), and trace quantities of fXa and thrombin sound the “alarm” for any potential dangers.

Circulating platelets contribute to localized thrombus formation at the site of vascular injury first by adhering to sub-endothelial collagen-vWF via their glycoprotein (GP) Ib receptors. Thrombin generated by TF-fVIIa/fXa (the “extrinsic pathway”) is capable of activating adherent platelets in its vicinity via protease-activated receptor 1 (PAR1) and PAR4. Thrombin-activated platelets play a pivotal role in subsequent coagulation processes in several ways. First, platelet GPIb receptors bind to fXI, and they also localize fIII to the site of endothelial disruption via its carrier protein vWF. Furthermore, partially activated fV is released from activated platelets. FXI, fVIII, and fV are involved in sustaining procoagulant responses (the “intrinsic pathway”) after thrombin-mediated activation. The serine protease fXIa mediates the activation of fIX to fIXa while fVIIIa serves as a cofactor to fIXa. FVa serves as a cofactor to fXa.

In clinical settings, the activated partial thromboplastin time (aPTT) [16], the prothrombin time (PT), along its international normalized ratio (INR) [17] assays are the three most widely used coagulation tests which correspond, respectively, to the intrinsic (fVIII, fIX, fXII, fXI, fV, fX, prothrombin, and fibrinogen) and extrinsic (prothrombin, fibrinogen, fV, fVII, and fX) pathways of the classical cascade model of hemostasis and measure the levels of individual coagulation factors in these pathways. In contrast, a thrombin time (TT) test measures the rate of the conversion of fibrinogen to fibrin and subsequent clot formation following the addition of thrombin to platelet-poor plasma samples [19]. The TT test may be used to diagnose fibrinogen abnormality when used in conjunction with a prolonged aPTT or PT finding, although it is not recommended for use as a stand-alone diagnostic test [19,20]. While having the advantages of being inexpensive, simple to perform, and easy to automate and validate, it is widely accepted that the aPTT, PT, and TT tests only partially reflect coagulation in a non-physiologic environment and do not capture any temporal or spatial contribution from specific cellular components fundamental to hemostasis [21,22]. Furthermore, the aPTT and PT assays only minimally reflect the levels of natural anticoagulants (e.g., TFPI, AT, and protein C/S) and cannot assess processes such as fibrin polymerization or clot stabilization that occur after the termination stage of these assays [23]. As such, these tests provide information on the initiation of clotting, but not hemostatic capacity in terms of final clot formation and the in vivo hemostatic process.

Apart from PT, aPTT is the most common coagulation test procedure performed in routine laboratories, useful for predicting the propensity for bleeding. The test is traditionally used for identifying quantitative and qualitative abnormalities in the intrinsic (factors VIII, IX, and XI) and common (factors II, V, and X) pathways of coagulation. Short aPTTs used to be dismissed as a laboratory artifact [47], but growing evidence suggests that these are associated with hypercoagulability, manifested as increased thrombin generation [48], and elevated procoagulant factors [49]. Short aPTTs are now considered to represent a procoagulant milieu [50] and have been shown to be associated with venous thromboembolism independent of other risk factors [51]. In patients with chest pain, short aPTTs are associated with increased risk of acute myocardial infarction [52]. They are also associated with impaired fasting glucose level and diabetics [53]. Short aPTTs, along with elevated fibrinogen levels, have also been found to be associated with diabetes [54].

## 3. Role of Platelets in the Pathogenesis of Adenomyosis

As of now, there are two prevailing theories on the pathogenesis of adenomyosis: metaplasia and invagination [55,56,57]. The former theory posits that the endometrial cells in the muscular layer originate from the metaplasia of Müllerian remnants or stem cells [58,59,60]. In contrast, the latter theory postulates that the direct invasion of endometrium to the muscle layer results from what is called tissue injury and repair that leads to the establishment of lesions [61,62,63]. One important foundation of the invagination theory is the tissue injury and repair (TIAR) hypothesis, proposed by Leyendecker and his associates [61,62], which postulates that injury induced by uterine hyperperistalsis/dysperistalsis causes adenomyosis. The TIAR hypothesis has recently been expanded to lump endometriosis and adenomyosis together as one disease, called archimetriosis [64].

Both theories make a lot of sense. However, so far, there has been no experimental data to support or refute either of the theories or the TIAR hypothesis [65,66]. Neither theory is seemingly backed by any epidemiological data. For the invagination theory in particular, it is unclear as to why and how the hyperperistalsis or injury—arguably the *primum movens*—occurs. Indeed, uterine peristalsis occurs in all women of reproductive age, but why is there only a fraction of them who develop adenomyosis? What are the risk factors, if any, for this hyperperistalsis/dysperistalsis? Is it possible to intervene or to forestall it? What can be done to mitigate the risk of hyperperistalsis in the first place? It is also unclear why and how the stem cells are recruited and then turned into endometrial epithelial and stromal cells that respond to hormonal fluctuations and become adenomyotic lesions.

In view of extensive epidemiological reports that iatrogenic uterine procedures, such as dilatation and curettage and induced abortion, increase the risk of adenomyosis [67,68,69,70,71], a new hypothesis, termed endometrial-myometrial interface (EMI) disruption (EMID), has been proposed recently [65]. Subsequent animal experiments demonstrate that both mechanically and thermally induced EMID can and does cause adenomyosis in mice [72]. More remarkably, the EMID hypothesis successfully predicted that the risk of developing adenomyosis depends on the mode and severity of EMID and that the risk can be mitigated by perioperative intervention [73]. It also gives a nice explanation as to why iatrogenic uterine procedures are a risk factor for adenomyosis. Of note, the EMID hypothesis also has been validated independently by another mouse model [74].

Tissue injury unavoidably causes disruption of local vasculature as well as the extravasation of blood, leading to platelet aggregation and the formation of clots. Vascular damage results in the loss of perfusion and consequent hypoxia. The tissue hypoxia can be further exacerbated by an influx of inflammatory and stromal cells—all with high metabolic demands for oxygen, which is essential for all aerobic organisms to produce energy via mitochondrial oxidative respiration and to perform other vital biological functions [75]. Once hypoxic, hypoxia-inducible factors (HIFs) are activated [76].

Conceivably, platelets play an important role in the EMID-induced adenomyosis. This is because EMID, induced either mechanically or thermally, would cause tissue and vasculature injury and, as such, platelet aggregation. Yet activated platelets alone can activate HIF-1α—the master regulator of hypoxia, effectively inducing a hypoxic sate in both endometriotic and endometrial stromal cells [77]. In addition, platelets increase the estrogen production in endometriotic stromal cells through upregulation of StAR, HSD3B2, aromatase, and HSD17B1 through the activation of NF-κB and/or TGF-β1 [78]. The increased local estrogen production may facilitate epithelial-mesenchymal transition (EMT), resulting in the invasion of endometrial epithelial cells into the myometrium through disrupted and thus compromised EMI [79].

Interestingly, estrogen has been well documented to be actively involved in tissue repair [80,81]. Numerous studies have shown that estrogen deficiency delays or impairs tissue repair [82,83,84,85,86]. In fact, estrogen is found to be involved in all phases of wound healing [80]. Hence the increased local production of estrogens in adenomyotic lesions [10] may merely reflect the fact that the lesions are indeed wounds.

Consequent to vascular damage, activated platelets can also release an array of cellular growth and angiogenic factors such as PDGF and VEGF, as well as inflammatory mediators, such as IL-8 and IL-1β [87,88,89]. Yet IL-1β released by activated platelets may help to induce de-differentiation of Schwann cells in the EMI region [90]. De-differentiated Schwann cells have recently been implicated in the genesis of adenomyosis induced by EMID [91].

## 4. Adenomyotic Lesions as Wounds

Like endometriosis, adenomyosis is viewed as an estrogen-dependent disease, characterized by the increased local production of estrogens due to molecular aberrations in steroidogenesis and estrogen-dependent growth of adenomyotic lesions [10]. It also has been recognized as a pelvic inflammatory condition, as manifested by elevated IL-1β, CRH and UCN in adenomyotic nodules [92], NF-κB activation [93,94,95], and the infiltration of macrophages and lymphocytes [96]. In fact, inflammation and coagulation are intricately coupled. Inflammation can activate the coagulation cascade, while coagulation modulates and sustains the inflammatory activity [97,98], establishing a mutually promotional loop. As enormously abundant hematopoietic cells that outnumber leukocytes in the peripheral [99], platelets are now viewed as inflammatory effector cells involved in the activities across the spectrum from acute inflammation to adaptive immunity [100,101]. Consequently, activated platelets are found to play a critical role in initiating inflammation [102]. In some diseases, such as rheumatoid arthritis, platelets can amplify inflammation through collagen-dependent production and release of microparticles [103].

Since ectopic endometrium experiences cyclic bleeding and thus ReTIAR [25], it resembles uncannily to wound healing. This is especially true for endometriotic lesions since scanty research has been done in this regard for adenomyosis. Similar to the inflammation phase in tissue repair, one study published in 2006 reported the rise of neutrophils, and then macrophages in surgically induced endometriosis [104]. In addition, as in tissue repair some immediate early and other genes such as c-Fos are activated in response to SRF [34], lesional expression of c-Fos also has been reported to be elevated at three months after induction of endometriosis in baboons but gradually tapered down later [105].

NETosis is known to play a role in wound healing [106], and NETs have been shown to promote fibroblast-to-myofibroblast transdifferentiation (FMT) and fibrogenesis [107]. This seems to account for elevated plasma NETs in women with endometriosis, especially with deep endometriosis [108]. While elevated NETs have not been reported in adenomyosis, they are very likely to be so. In addition, many DAMP molecules, which are released upon tissue injury or cellular stress and are regarded as endogenous danger signals, such as fibrinogen and two members of the alarmin family, galectins and annexins [109,110], which have also been found to be elevated in adenomyosis [111,112,113,114,115].

In both endometriosis and adenomyosis, EMT has been well recognized to play an important role [79,116,117]. However, EMT is well known to be actively involved in tissue repair as well as fibrosis [118]. Similarly, it has been reported in wound healing that the elevated expression of the CD47—as a “don’t eat me” signal [119]—on murine fibroblasts protect them from being phagocytized and eliminated by macrophages [120], leading to excessive matrix deposition. CD47 interacts with signal regulatory protein α (SIRPα) and regulates the disposal of ineffective normal cells [121]. Thrombospondin-1 (TSP-1) is released by activated platelets and is a ligand of CD47 [122]. TSP-1 also has been shown to participate in fibrogenesis [123].

In endometriosis, the lesional staining of CD47 is indeed elevated and reduces the phagocytosis efficiency of macrophages on endometriotic stromal cells [124]. In addition, TSP-1/CD47/SIRPα collectively enhances cellular viability, reduces apoptosis, and facilitates fibrosis [124]. On the other hand, blocking CD47 ameliorates endometriosis [125]. These findings are consistent with the known role of platelets in tissue repair and are likely to also hold true for adenomyosis.

Yet elevated CD47 expression, along with the activation of Akt, in fibroblasts is a common feature of many fibrotic conditions, induced by the activation of AP1 transcription factor c-Jun in the pathologic fibroblasts [120]. Uncannily similar, both c-Jun and Akt activation have been reported in endometriosis [126,127,128,129]. While c-Jun activation has not been reported in adenomyosis as of now, the activation of Akt has [130]. Consistently, the immunostaining of PP2A, the negative regulator of Akt and of PTEN, and the negative regulator of the PI3K/Akt signaling pathway also have been reported to be reduced in adenomyosis [131].

The α-granules of platelets contain high mobility group box 1 (HMGB1), which is released when platelets are activated [36]. As one of the alarmin/DAMP molecules, elevated plasma HMGB1 levels and lesional expression have been reported in endometriosis [132,133]. It is very likely to be elevated in adenomyotic lesions as well.

In view of the above, it can be seen that adenomyotic lesions are indeed similar to wounds, just as endometriotic lesions. In fact, the role of platelets in the development of adenomyosis has been gradually revealed and recognized in the last few years [134,135], starting with the realization that adenomyotic lesions, just like their endometriotic counterpart [136,137], are practically and fundamentally wounds undergoing ReTIAR [25].

In tissue repair, it is well documented that estrogen is actively involved [80,81]. In fact, estrogen has been shown to be vital to tissue repair, and its deficiency delays or impairs repair [82,83,84,85,86]. Estrogen has been reported to be involved in all phases of tissue repair [80]. Remarkably, endometriotic stromal cells co-cultured with activated platelets display upregulated ERβ [138]. Consistent with the notion that ERβ is shown to play a critical role in tissue repair [139,140], ERβ is overexpressed in adenomyotic lesions [141], as in endometriotic lesions [142,143].

Platelet α-granules contain loads of TSP-1 [144], which are released upon platelet activation. Therefore, the elevated CD47 expression in ectopic endometrium is very likely due, at least in part, to platelet-derived TSP-1. While CD47 overexpression has not been reported in adenomyosis, it is highly likely to be so. Platelets may also be responsible for an increased local production of estrogen and elevated ERβ expression in adenomyotic lesions.

**Figure 1:**
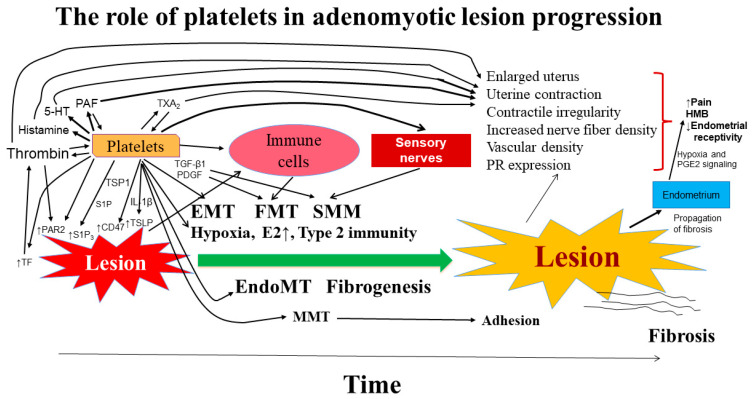
Schematic illustration of the roles of platelets in the progression of adenomyotic lesions. The arrows indicate the crosstalk between adenomyotic lesions and other cells in the lesional microenvironment. Many cells in the lesional microenvironment interact with adenomyotic lesions, and cytokines/chemokines, growth factors, and neuropeptides released by these cells, such as platelets (anuclear), immune cells (such as macrophages and lymphocytes), and sensory nerve fibers can accelerate the development of adenomyotic lesions. Type 2 immunity also promotes fibrogenesis of adenomyotic lesions. Histamine, 5-HT, PAF, TXA_2_ and thrombin derived from activated platelets may also enhance uterine contractility. The upward arrows indicate upregulation/overexpression or increase. The directional arrows mean “lead to” or “result in”. Abbreviations used: 5-HT = 5-hydroxytryptamine, serotonin; E2 = 17β-estradiol; EMT = epithelial–mesenchymal transition; EndoMT = endothelial-mesenchymal transition; FMT = fibroblast-to-myofibroblast transdifferentiation; HMB = heavy menstrual bleeding; MMT = mesothelial-mesenchymal transition; PR = progesterone receptor; SMM = smooth muscle metaplasia; PAF = platelet activating factor; PAR2 = protease activated receptor 2; PGE2 = prostaglandin E2; S1P = sphingosine 1-phosphate; S1P_3_= sphingosine 1-phosphate receptor 3; PDGF = platelet-derived growth factor; TGF-β1 = transforming growth factor β1; TXA2 = thromboxane A2; TSLP = thymic stromal lymphopoietin; TSP1 = thrombospondin-1; TF = tissue factor.

While the role of EMT in facilitating invasion in adenomyosis has been well-documented, few, if any, studies have raised the question of why EMT is involved in the first place. Yet from the ReTIAR perspective, this is actually something to be expected since EMT is known to be vital in wound healing as it is rapidly activated and results in wound closure through re-epithelialization [145], aside from its involvement in development and cancer metastasis. Similarly, myofibroblast activation or FMT is also expected since it leads to tissue contraction, restoring tissue integrity and reducing the wound size [146]. When the repair process goes awry, as in the case of chronic inflammation or recurrent/repeated tissue injury as in adenomyotic lesions, fibrogenesis ensues through prolonged inflammation, EMT, and FMT [147]. Figure 1 presents a schematic diagram that depicts the roles of platelets in adenomyosis [10,35,58,77,78,80,81,134,135,136,137,138,140,141,148,149,150,151,152,153,154,155,156,157,158,159,160,161,162,163,164,164,165,166,167,168,169,170,171,172,173,174,175].

## 5. Platelets Promote Progression of Adenomyotic Lesions

With the understanding of the natural history of endometriotic lesions in the last few years [25], we now have a better understanding of the natural history of adenomyotic lesions, which are nearly identical to that of endometriosis. Since adenomyotic lesions undergo cyclic bleeding just as eutopic endometrium, they are fundamentally wounds undergoing ReTIAR just like endometriotic lesions [25]. As such, they undergo EMT, FMT, and smooth muscle metaplasia (SMM) and progress ultimately to fibrosis [134,135,136]. Due to FMT and SMM, stromal cells within lesions eventually are differentiated into smooth muscle cells (SMCs) [134,135,136], which are presumably fused into existing myometrium, causing enlarged uterus in women with adenomyosis. Myometrial SMCs from women with adenomyosis may also experience cellular hypertrophy [176], likely due to the activation of the MAPK/ERK and the PI3K/mTOR/Akt pathways [177] and the upregulation of cannabinoid receptor CB1 [178]. Regardless of the source, the resultant enlarged uterus would exhibit increased magnitude and/or frequency of uterine contraction, particularly when oxytocin receptor (OTR) is overexpressed [179]. Additionally, due to the fusion—by no means seamlessly—of newly turned SMCs into the myometrium, the uterine contraction is likely to be out of synchronization [180], resulting possibly in spasm-like contractions.

One strong indication for the involvement of platelets in adenomyosis is the increased immunostaining of TF in both eutopic and ectopic endometrium from women with adenomyosis [181]. As a cellular receptor, TF binds fVII/fVIIa to initiate the coagulation cascade, even though its role in adenomyosis-induced HMB was thought to be of angiogenic in nature [181]. In particular, higher TF staining in eutopic endometrium is found to be associated with the HMB [181]. TF levels in endometrial bleeding sites have been reported to be higher than that in the non-bleeding ones [182], accompanied by abnormally enlarged and distended blood vessels in the bleeding sites suggestive of an angiogenic role of TF [183]. Moreover, lesional staining of plasminogen activator (PA) inhibitor 1 (PAI-1), which inhibits PA, is reported to be elevated in adenomyosis [184]. This may suggest an impaired fibrinolytic system in adenomyosis.

Thrombin can activate proteinase-activated receptors (PARs), which are members of the G protein-coupled receptor (GPCR) family. The PAR family has four members: PAR-1, PAR-2, PAR-3, and PAR-4 [185]. PAR activation induces the activation of mitogen-activated protein kinases (MAPKs), including extracellular signal-regulated kinases (ERK1/2), c-Jun N-terminal kinases (JNKs), and p38 MAPK. PAR-1 and PAR-2 are expressed in eutopic and ectopic endometrium from women with endometriosis [185].

Similar to adenomyosis [181], TF is upregulated in endometriosis as well [186]. Both PAR-1 and PAR-2 have been reported to be expressed in endometriotic stromal cells [186,187,188]. Thrombin stimulation increases the gene expression and secretion of IL-8 and MCP-1 from endometriotic stromal cells, and PAR-1 activation also induces TF expression [187]. In addition, IL-8 also upregulates TF in endometriotic stromal cells [187]. Thus, it seems that a feed-forward loop exists, which promotes inflammation and coagulation back and forth in endometriosis and likely also in adenomyosis.

PAR-2 in endometriotic stromal cells can be activated by the TF-fVIIa complex and the TF-fVIIa-fXa complex, but it also can be activated by mast cell-derived tryptase, which appears to be aplenty as the number of mast cells is increased in endometriosis [189,190]. In addition, it can be induced by TGF-β1 in endometriosis [191]. PAR-2 activation in endometriotic stromal cells induces increased secretion of IL-6 and IL-8 and stimulates cellular proliferation [188]. While neither PAR-1 nor PAR-2 has been reported in adenomyosis so far, given the commonality that both adenomyotic and endometriotic lesions share, it is highly likely that they also should play a role in adenomyosis.

Since activated platelets release copious amount of TGF-β1 [192], which is a prototypical factor in EMT [193], FMT [194,195], and subsequent fibrogenesis [196], platelets can and have been shown to induce EMT, FMT, and SMM in adenomyosis, promoting fibrogenesis [134,135,136]. Aside from adenomyotic cells, many other cells within lesions and the uterus may also participate in the progression of adenomyosis. Stimulated by activated platelets, vascular endothelial cells can be differentiated into myofibroblasts within lesions through endothelial-mesenchymal transition (EndoMT) [166]. Activated platelets can also turn peritoneal mesothelial cells into myofibroblasts through mesothelial-mesenchymal transition (MMT) [197], causing adhesion and the immobility of the uterus in women with adenomyosis. Moreover, macrophages within adenomyotic lesions would interact with adenomyotic cells and become polarized into alternatively activated macrophages (M2 macrophages), and, along with regulatory T cells (Tregs) within lesions, promote lesional fibrogenesis through type II immunity [168,169].

Platelets may also induce the lesional expression of thymic stromal lymphopoietin (TSLP) through platelet-derived IL-1β [198,199] and drive type 2 immunity that facilitates lesional fibrogenesis [169,198], similar to how platelets induces endothelial TSLP expression to promote fibrogenesis in human systemic sclerosis [199]. Consistent with platelet-induced CD69+ T cell activation [45], there is an increase in CD69+ T cells in the peritoneal fluid from women with endometriosis [200,201] and possibly in adenomyosis as well.

### 5.1. Platelet-Derived Growth Factors

Platelet-derived PDGF, which has been shown to be elevated in endometrial tissues and in the peritoneal fluid of women with endometriosis [202,203,204] and possibly with adenomyosis, are reported to stimulate endometrial cell proliferation, invasiveness, and migration [202,205,206,207]. However, PDGF has been well-documented to be a pro-fibrotic factor [208,209] and has also been implicated in fibrogenesis of endometriosis [169] and likely of adenomyosis as well.

### 5.2. Serotonin

Activated platelets also release serotonin (5-hydroxytryptamine, or 5-HT), a molecule with pleiotropic functions and also a powerful vasoconstrictor. While its role in adenomyosis or endometriosis has not been fully understood, its pro-fibrotic role suggests that it may play a role in adenomyosis. One notable example for the link between 5-HT and fibrotic diseases is the condition called carcinoid syndrome, which is characterized by tissue fibrosis in various organs and caused by neuroendocrine carcinoid tumors that secrete vast quantities of 5-HT [210]. It also has been reported that 5-HT facilitated collagen production in fibroblasts through 5-HT_2B_ serotonin receptor in a TGF-β-dependent manner [211]. In addition, 5-HT receptor inhibitors stalled fibrogenesis in mouse models of fibrosis, and platelet inhibition led to decreased 5-HT content in the fibrotic skin and decreased dermal thickening in mice [211]. Serotonin also has been shown to aggravate bleomycin-induced pulmonary fibrosis in mice through promoting inflammation, exudation of proteins and cells, oxidative stress, and upregulation of fibrosis-associated genes in the lung tissues [212]. Antagonism of serotonin receptors 5-HT_2_ and 5-HT_2B_ is shown to attenuate pro-fibrotic phenotype in human adult dermal fibroblasts by blocking TGF-β1 induced non-canonical signaling pathways including STAT3 and ERK1/2 [213]. Antagonism of 5-HT_2B_ also has been shown to hinder TGF-β1-induced valvular myofibroblast differentiation through inhibition of p38 MAPK phosphorylation [214] and also to attenuate myofibroblast differentiation and subsequent fibrotic responses in vitro and in vivo [215]. Whether platelet-derived 5-HT has any pro-fibrogenic role in adenomyosis thus warrants further investigation.

### 5.3. Conflicting Findings

A recent study found no evidence of platelet aggregation in adenomyotic lesions [216], which is directly at odds with those reported in [134,135]. Yet a close look at the study reveals that the choice of both cases and controls may be the source of the discrepancy. First, the study recruited 17 patients with exclusively severe diffuse adenomyosis who underwent hysterectomy. Apparently, the disease condition was severe enough for these patients to undergo hysterectomy. In other words, the adenomyotic lesions in these patients were well established and, as such, highly fibrotic. As shown previously, ectopic endometrium with higher fibrotic content has significantly less platelet aggregation than that of lower fibrotic content, due possibly to reduced vascularity [217]. Hence adenomyotic lesions in these patients may have had less platelet aggregation than those of early-stage lesions.

For controls, the study used endometrial tissue samples “from 23 patients undergoing hysterectomy (17%), hysteroscopic biopsy (35%) or curettage (48%). Hysterectomy was carried out due to intramural myoma and none of the patients was diagnosed with adenomyosis or endometriosis. The purpose of intervention by biopsy or curettage was diagnostic in the context of infertility (50%) or heavy menstrual bleeding (50%)” [216]. In contrast, the control sample from [134] consisted of endometrial tissue samples through curettage from 20 women with teratoma (*n* = 1, 5%), cervical intraepithelial neoplasia (CIN)-III (*n* = 14, 70%), stage Ia1 cervical cancer (*n* = 3, 15%), and cervical carcinoma in situ (*n* = 2, 10%), but without any clinical indication or history of adenomyosis or endometriosis. The choice of controls was not perfect, but effectively minimized the risk of having a coagulant endometrium.

While it is understandable that endometrial samples were collected from those who sought medical attention, it is also notable that the majority of the controls (*n* = 19, or 83%) had either uterine fibroids or problems in fertility or HMB. It is known from the PALM-COIEN classification that coagulopathy is one possible cause for HMB (AUB-C). As such, it is possible that the control samples could also have the tendency of having increased platelet aggregation. Consequently, the tendency of less, but still existent, platelet aggregation in cases coupled with possible involvement in coagulation in controls (fibroids and coagulopathy) may have significantly reduced the signal-to-noise ratio, obscuring the real difference.

The confidence on the role of platelets in adenomyosis can be further bolstered by numerous case reports documenting thromboembolism in women with adenomyosis [28], along with the overexpression of TF in adenomyosis [181]. Additionally, a recent study reported that, compared with women without adenomyosis, women with adenomyosis had higher platelet count and shorter TT and aPTT [218], and another study reported shorter PT and the negative correlation between uterine size and aPTT/TT [111], suggesting that adenomyosis is associated with hypercoagulability. Our recent data also demonstrates that indeed women with adenomyosis who experienced HMB are also in a hypercoagulable state as in endometriosis (Liu et al., submitted for publication).

### 5.4. Platelet Activation by Thrombin/Thromboxane

While cyclic bleeding [24] inevitably leads to platelet aggregation, there is also evidence for extravasated platelets in endometriosis [151] and possibly in adenomyosis as well. This seems to suggest that the relationship between adenomyotic lesions and platelets are not entirely uni-directional, but, rather, bi-directional. In fact, it has been reported that increased production of thromboxane B2 (TXB_2_), a metabolite of TXA_2_, by endometriotic stromal cells stimulated with IL-1β, increases TF expression as well as thrombin concentration in peritoneal fluids from women with endometriosis [151], suggesting that endometriotic and perhaps adenomyotic lesion as well and its microenvironment are conducive to platelet activation and aggregation.

Indeed, endometriotic stromal cells secrete thrombin and TXA_2_ and induce platelet activation in a density-dependent fashion [219]. Specifically, co-culture of platelets with endometriotic stromal cells results in increased concentration of TXB_2_, thrombin, and TGF-β1 in a density-dependent manner [219]. Treatment of endometriotic stromal cells with hirudin (a specific thrombin inhibitor) and Ozagrel (a TXA_2_ synthetase inhibitor), but not apyrase (an adenosine diphosphate (ATP) pathway inhibitor), resulted in significant and substantial suppression of platelet aggregation [219]. Since adenomyotic stromal cells are very similar to their endometriotic counterpart and share many molecular aberrations [93], there is reason to believe that adenomyotic stromal cells would behave similarly and secrete platelet-activating molecules, such as thrombin and TXA_2._ Thus, platelets and adenomyotic lesions are likely to engage crosstalk, collectively facilitating the progression of adenomyotic lesions and induce a hypercoagulable state in patients with adenomyosis.

### 5.5. Platelets-Mediated Suppression of Cytotoxicity in NK Cells in Ectopic Endometrium

As a key component of the innate immune system, natural killer (NK) cells are a subset of lymphocytes that provide the first-line defense against pathogens or transformed cells by exerting cytotoxicity and the regulation of cytokine producing effector functions [220,221]. The function of NK cells is tightly regulated by an array of functionally opposing surface receptors, inhibitory receptors that bind major histocompatibility complex (MHC) class I molecules and protect “self”, and activating receptors that bind ligands on virus-infected or tumor cells [222]. Activating and inhibitory receptors can transduce, respectively, positive or negative signals to regulate NK cell cytotoxicity and cytokine release [223]. NK cells may play an important role in peritoneal immune surveillance, possibly eliminating ectopic endometrial cells, with low or absent expression of MHC class I and stress-induced expression for activating NK receptors in women without adenomyosis. However, the role of NK cells in adenomyosis has so far been inconclusive [224].

However, activated platelets within adenomyotic lesions could provide a physical shield to adenomyotic cells, protecting them from cytotoxicity rendered by NK cells as in endometriosis [225]. In fact, platelet coating, as could happen following cyclic bleeding, provides ectopic endometrial cells a physical cloak against NK cells as well as increased MHC-I expression, effectively providing a cloak of “pseudo-self” to coated cells to protect against NK cell lysis [225]. Co-incubation of target cells with platelets reduces the expression of NKG2D ligands MICA and MICB and reduces the NK cell cytotoxicity. In addition, co-incubation of NK cells with platelets also impairs the NK cell cytotoxicity, and this impaired NK cell cytotoxicity is not due to the increased NK cell apoptosis, but, rather, through reduced NK cell degranulation and IFN-γ production, the reduced expression of activating receptors NKG2D and NKp46, and the increased expression of inhibitory receptor KIR2DL1 in NK cells [225]. On the other hand, TGF-β1 neutralization abolishes the aberrant expression of NKG2D, NKp46, and KIR2DL1 and partially restores the impaired NK cell cytotoxicity induced by activated platelets and their releasate [225]. Taken together, these data provide a strong piece of evidence that activated platelets, which are aggregated in ectopic endometrium following cyclic bleeding or simply due to the release of platelet-activating molecules by endometriotic stromal cells [219], impair NK cell cytotoxicity in endometriosis through multiple mechanisms and both soluble and membrane-bound factors are required for NK cell evasion of endometriotic cells. Platelet-derived TGF-β1 may reduce the expression of the activating receptor NKG2D as well as cytotoxicity of NK cells in women with adenomyosis, as in endometriosis [226].

### 5.6. Platelets and Coagulation in Adenomyosis-Induced HMB

It has been well-documented that HMB results from impaired endometrial repair [27,227]. As such, platelets and/or coagulation must be involved in endometrial repair and thus HMB. In fact, coagulopathy is one known cause for AUB and has been designated in the FIGO PALM-COIEN classification [228].

Coagulation pathways should be and are involved in adenomyosis in general and adenomyosis-induced HMB (ADM-HMB) in particular for the following reasons. First, coagulation is set off by tissue injury, or more precisely, vascular injury. Platelets are known to play an important role in the development of adenomyosis [134,135]. In fact, anti-platelet therapy has been shown to have therapeutic effect in mouse models of adenomyosis [229], similar to that in endometriosis [230,231]. Thus, the hypercoagulation in adenomyosis agrees with the aggravation and activation of platelets within adenomyotic lesions, which, in turn, induce PAI-1 expression in ectopic endometrium [136], as shown in adenomyosis [184].

Second, extrinsic compression of blood vessels by an enlarged uterus may cause venous stenosis and thus stasis, as well as hypercoagulability, as shown in this study. Adenomyotic stromal cells may also secrete thrombin and TXA_2_, similar to endometriotic stromal cells [219]. Thus, a positive feed-forward loop could be established, contributing to the hypercoagulability in women with adenomyosis [218], especially in those who complained of HMB. In addition, patients with adenomyosis often have elevated CA125 levels, especially those with a large uterus [232]. High CA125 levels may be associated with cerebral infarction [233], and they correlate with D-dimer levels under a high tumor burden [234]. Furthermore, red blood cells may also be caught in the process of thrombus formation and contribute to the mass of the thrombus; as such, severe anemia might impair normal hemostasis. This would suggest that patients who take iron supplements may have shorter aPTT and higher plasma FDP levels than those without.

Third, irrespective of the cause, HMB ultimately results from impaired endometrial repair [235], which is intricately linked with coagulation as well as fibrinolysis. It is not surprising that coagulopathy is one of the causes for AUB (AUB-C). In fact, the stoppage of menstrual bleed loss (MBL) can be attained through endometrial hemostasis by platelet aggregation, fibrin deposition, and thrombus formation, which are regulated at the molecular level by coordinated and intricate interactions of hormonal, immunological, and hemostatic factors.

Patients with adenomyosis who were experiencing HMB had a significantly higher platelet count and significantly higher plasma fibrinogen and D-dimer levels, but they had shorter PT and aPTT as compared with women without adenomyosis (Liu et al., submitted for publication). Within the ADM-HMB patients, patients who complained of MBL of 100 mL or higher had significantly higher plasma D-dimer and fibrin-degradation products (FDP) levels but shorter aPTT and lower plasma fibrinogen levels than those who had MBL of less than 100 mL (Liu et al., submitted for publication). These data strongly suggest that women with adenomyosis who complained of HMB are in a hypercoagulable state as compared with women without adenomyosis, more so in those who complained of excessive MBL.

APTT and PT are often used to evaluate the intrinsic and extrinsic pathways of coagulation, respectively, both in conjunction with the common pathway [236]. Hence, these finding suggests that, compared with women without adenomyosis, the enhanced hypercoagulation in adenomyosis [218] and women with adenomyosis who complained of HMB may be attributable to the activation of *both* intrinsic and extrinsic coagulation pathways. Fibrinogen is a known coagulation factor associated with hypercoagulation [237] and the release of TXA_2_. Once thrombin is generated, fibrinogen activates platelets to produce TXA_2_, resulting in more platelet activation, which, in turn, induces PAI-1 expression in ectopic endometrium [136], as demonstrated recently in adenomyosis [184]. This may establish a vicious cycle in maintaining platelet activation, the activation of the coagulation cascade, and then higher plasma fibrinogen levels and shorter aPTTs in adenomyosis.

The elevated fibrinogen levels in ADM-HMB patients as compared with women without adenomyosis (Liu et al., submitted for publication) is suggestive of a heightened coagulable state in the former group. In contrast, within ADM-HMB patients who experienced excessive HMB were found to have *decreased* fibrinogen levels as compared with those of moderate-heavy MBL. Since thrombin converts soluble plasma fibrinogen into fibrin, the reduced fibrinogen levels may suggest a more efficient conversion of fibrinogen to fibrin, thus increased fibrinolytic activity probably due to the increased thrombin production, since thrombin acts as a serine protease that converts soluble fibrinogen into insoluble strands of fibrin, as well as catalyzing many other coagulation-related reactions [238].

These notions are consistent with the report that both eutopic and ectopic endometrium in women with adenomyosis exhibit elevated expression of TF and that the TF immunoreactivity in eutopic endometrium correlated positively with the amount of MBL [181]. Adenomyotic lesions may also release TF-positive macrovesicles, which may lead to the direct initiation of the coagulation cascade since TF activates the extrinsic pathway of the coagulation cascade, resulting in thrombin generation, platelet aggregation, and clot formation. These findings also are consistent with numerous case reports documenting increased thrombotic propensity in women with adenomyosis [28,29,30,31,32].

TF activation increases the production of thrombin, which is known to induce VEGF expression in several cell types [239,240] and to induce VEGF secretion from human endometrial stromal cells undergoing decidualization [241]. Since VEGF overexpression also results in vascular permeability, persistent thrombin generation could lead to enlarged, fragile, and leaky vessels, overwhelming TF-thrombin mediated hemostasis and, as such, causing HMB. This is especially true since thrombin and a PAR-1 agonist increase the production of MMP-1 and active MMP-2 in endometrial stromal cells [242], raising the possibility that thrombin may facilitate tissue degradation or impair endometrial integrity via PAR-1, increasing the amount of MBL.

There are several implications. First, the realization of the hypercoagulability in patients with adenomyosis who complained of HMB should help healthcare providers to take precautionary or even prophylactic measures when seeing such patients due to increased risk of thrombotic events [28,29,30,31,32]. In addition, the conceivable increase in thrombin levels in adenomyosis patients who complained of excessive HMB may also signal possible impaired decidualization since thrombin can reduce the secretion of prolactin, a key marker of decidualization, alter the morphological transformation of decidualizing endometrial stromal cells, and activate genes involved in matrix degradation and proinflammatory chemokines [243]. Moreover, the hypercoagulability in these patients, the heightened coagulation and fibrinolytic activity in adenomyosis who complained of excessive HMB s, along with the anticipated alleviation of hypercoagulation after removal or perhaps containment of adenomyotic lesions as in endometriosis [244], suggest that coagulation parameters could be used for monitoring the treatment response/efficacy.

## 6. Platelets and Coagulation in Adenomyosis-Induced Dysmenorrhea

As of now, the molecular mechanisms underlying adenomyosis-induced dysmenorrhea are not fully elucidated, but they are thought to be attributable to increased uterine contractility, hyperinnervation, increased lesion-derived pain mediators, and central sensitization. Through platelet-derived TGF-β1, activated platelets can promote the progression of adenomyosis through EMT, FMT, SMM and fibrogenesis [134,135] as well as EndoMT and MMT [166,197]. The increasing lesional fibrosis is correlated with the severity of dysmenorrhea [245,246]. Platelet activation and the resultant induction of TF in adenomyotic lesions [93,181] would result in the release of thrombin in the lesion and then to its neighboring myometrium, which may facilitate, through PAR-1 [247,248], uterine contractions [249,250], contributing to dysmenorrhea.

In addition, histamine and serotonin released by platelet dense granules when activated have been shown to increase uterine contractility [251,252,253,254]. As adenomyotic lesions are located within the myometrium and platelet-derived histamine and serotonin are likely to permeate into neighboring myometrium, eliciting increased uterine contractility and, as such, contributing to dysmenorrhea.

Activated platelets also release platelet-activating factor (PAF) [144], which may be involved in dysmenorrhea and pelvic pain [255]. Indeed, in women with endometriosis, a condition associated with NSAID-resistant dysmenorrhea, peritoneal PAF synthesis is enhanced [256]. In contrast, PAF acetylhydrolase, which hydrolyzes PAF and related oxidized phospholipids, is reported to be reduced in the peritoneal fluid [257]. Intraperitoneal injections of a PAF receptor agonist CPAF and PGF_2α_ evoked visceral pain and pelvic hyperalgesia through reducing uterine perfusion and increased uterine contractility [163,164,258,259,260,261,262,263,264,265,266,267,268].

## 7. Putting Pieces Together

The adoption of the view that adenomyotic lesions are wounds undergoing ReTIAR has important implications for both research and clinical management of adenomyosis. It immediately provides a broader perspective on the big picture of the natural history of adenomyotic lesions. Several key molecular processes, such as EMT, FMT, SMM, and fibrogenesis, are now in plain sight, and with these we can see not just leaves, twigs, branches, and trees, but also the entire forest—in broad strokes, at the very least.

The ReTIAR framework also provides a dynamic view of the lesional progression and underscores the importance of lesional microenvironment (Figure 1). Indeed, many cells in adenomyotic lesions may change their identity—epithelial cells become mesenchymal cells and thus stromal/fibroblasts, and fibroblasts can be turned into myofibroblasts. Many cells, including platelets, within the lesional microenvironment are not merely innocent bystanders but are actually active aiders and abettors that co-conspire with adenomyotic cells to facilitate lesional progression.

The ReTIAR prism not only provides a much needed backbone with which we can tether to and piece together many seemingly unrelated findings, but also help to guide us to make useful predictions. For example, given the role of CD47 in tissue repair and in endometriosis, and particularly given that platelets, when activated, can release TSP-1 [144], we can deduce that platelet-derived TSP-1 can upregulate CD47 expression in adenomyotic lesions and reduce the phagocytosis efficiency of macrophages on adenomyotic stromal cells, as in endometriosis [124]. In addition, TSP-1/CD47/SIRPα collectively enhance cellular viability, reduce apoptosis, and facilitate fibrosis, as in endometriosis [124].

Platelet granules contain a variety of important adhesive, inflammatory, angiogenic, and pro-thrombogenic molecules [269], and their roles in the development of adenomyosis have not been investigated so far. Many other scattered and seemingly unrelated findings can actually be pieced together. For example, plasma levels of lysophosphatidic acid (LPA) have been reported to be elevated in women with adenomyosis, along with some of its receptors LPARs [270]. In endometriosis, it has been reported that LPA facilitates the invasiveness of endometriotic epithelial cells through LPAR1 and LPAR3 [271]. In adenomyosis, it has been reported recently that estrogen-increased SGK1 promotes endometrial stromal cell invasion by regulating LPAR2 [272], which also is shown to play a role in ovarian endometrioma [273]. However, activated platelets release LPA [144]. In addition, SGK1, which is a target of TGF-β1 [274], has been shown to be a powerful regulator of platelet dense granule biogenesis, platelet secretion, and thrombus formation [275]. In fact, platelet-derived LPA promotes proliferation in tumor cells [276].

Similarly, the sphingosine 1 phosphate (S1P) signaling pathway has been reported to be dysregulated in adenomyosis, manifesting as elevated S1P receptor S1P_3_ but not S1P_2_ expression [277]. In endometriosis, S1P also increased IL-6 expression and cellular proliferation [278] and induced alternatively activated macrophages [279]. In particular, several S1Ps are overexpressed in both ovarian endometrioma and deep endometriotic lesions and appear to be involved in TGF-β1-induced fibrogenesis [280]. Again, activated platelets release S1P [144], which can also induce TF expression in endothelial cells [281].

The dynamic progression of adenomyotic lesions would help us in the clinical management of adenomyosis. For example, with the understanding that the PGE_2_ signaling is important for early lesions but is detrimental to older lesions [282,283], one should refrain from the use of COX-2 inhibitors for treatment purposes for patients with well-established or advanced adenomyosis. In addition, one may capitalize on the knowledge of progressive fibrogenesis of adenomyosis and employ elastography to better diagnose adenomyosis [245].

## 8. Therapeutic Implications

In view of the facilitating role of platelets in adenomyosis progression, naturally, one may think that anti-platelet therapy can have therapeutic potentials. Remarkably, in traditional Chinese medicine (TCM), adenomyosis- or endometriosis-related symptoms such as dysmenorrhea, pain, and infertility have always been recognized as being caused by “blood stasis” or, in modern medical parlance, aberrant coagulation, and the treatment has been invariably the use of herbs, in various concoctions, that are now known to be anti-platelet or anti-thrombotic. Indeed, it has been reported that platelet depletion resulted in a significantly reduced lesion size and improved hyperalgesia in mice with induced endometriosis [151]. The treatment with a recombinant P-selectin in a mouse with induced endometriosis resulted in soluble P-selectin treatment markedly reduced the lesion size in the mouse through decreased platelet aggregation and angiogenesis, improved general hyperalgesia, and the reduced extent of macrophages infiltration, resulting in reduced fibrotic tissue content [230]. In addition, treatment with Ozagrel, a TXA_2_ synthase inhibitor, yields significant reduction in lesion growth along with improved hyperalgesia in mice with induced endometriosis [231]. Other anti-platelet compounds, such as scutellarin [284], andrographolide [285], and sodium tanshinone IIA [286], also show therapeutic potentials in preclinical studies [287,288]. Similar to endometriosis, the treatment with the anti-platelet drug Ozagrel, as well as platelet depletion, dose-dependently reduced platelet aggregation and the number of macrophages; suppressed myometrial infiltration; improved generalized hyperalgesia; reduced uterine contractility; lowered plasma corticosterone levels; and reduced the lesional staining of COX-2, phosphorylated NF-κB p65 subunit, oxytocin receptor, and TRPV1, but it elevated the lesional staining of PR-B and collagens and slowed down the process of fibrogenesis of mouse with induced adenomyosis [229]. Similarly, andrographolide [180], epigallocatechin-3-gallate [289], resveratrol [290], quercetin [291], leonurine [292], berberine [293], and valproic acid [180,294] all have been demonstrated to be therapeutic potentials in mice with induced adenomyosis. In addition, andrographolide [93], tanshinone IIA [295], and valproic acid [296] have demonstrated their therapeutic potential through in vitro studies.

Andrographolide [297], berberine [298], resveratrol [299], quercetin [300], leonurine [301], tanshinone IIA [302,303], valproic acid [304], and even danazol [305] are known to be anti-platelet. Consistent with the preclinical studies, valproic acid, andrographolide, and danazol ring have shown clinically to be promising in treating adenomyosis [306,307,308,309]. Other compounds have been tested in preclinical studies, but not in humans yet.

## 9. Summary and Perspective

Growing evidence indicates that, in a nutshell, adenomyotic lesions are fundamentally wounds undergoing ReTIAR due to cyclic bleeding. Platelets are rapidly deployed to and aggregate at the wounded site to initiate hemostasis, inaugurating the tissue repair process of inflammation, proliferation, and tissue remodeling [36]. Activated platelets secret a plethora of bioactive molecules, including various cytokines/chemokines and growth factors [192]. As such, the involvement of platelets in adenomyosis appears to be, in retrospect, rather obvious. Yet platelets do not just passively impact adenomyotic lesions. Adenomyotic stromal cells may also produce potent platelet-activating molecules, such as thrombin and TXA_2_ [219], and collagens [136], which, coupled with increased angiogenesis and thus vascular permeability, may further lead to platelet aggregation. Consequently, adenomyotic lesions and platelets engage active cross-talks to maintain lesion growth and facilitate lesional progression and fibrogenesis [136,137,151].

Because of the involvement of platelets and TF activation in adenomyosis, women with adenomyosis are in a hypercoagulable state [218], and this may account for the increased risk of thrombosis as reported in the literature [28]. This seems to suggest that adenomyotic lesions and platelet activation are mutually causative, or at least they are intimately entwined.

From the perspective ReTIAR, the natural history of adenomyotic lesions can be easily grasped, although a lot of details are still in need of ironing out. With this prism, we can see the important role of platelets and other immune cells in the development of adenomyosis, as well as adenomyosis-induced HMB and dysmenorrhea. Importantly, the notion of ReTIAR provides a much needed framework so that we can tether to and piece together many seemingly unrelated findings. Importantly, the ReTIAR also helps us to make useful predictions, for example, the role of platelet-derived TSP-1, PAF, HMGB1, and S1P, which otherwise seem to be utterly unrelated. More importantly, this ReTIAR notion also gives us clues to look for and round up other culprits that caused adenomyosis, which are otherwise unsuspected or overlooked, and, as such, at large. These would include, but not limited to, histamine, serotonin, and thrombin, which are very likely to be involved in the progression of adenomyosis and adenomyosis-induced HMB and/or dysmenorrhea. Of course, future studies are warranted to interrogate these suspects.

While the involvement of platelets in the progression of adenomyosis is gaining support, their roles in interacting with other immune cells in the context of lesional progression are still poorly understood. For example, platelets seem to work with Treg cells to form a type 2 immunity in lesional microenvironment that is conducive to lesional progression and fibrogenesis [169]. In other words, we have just scratched the surface. How platelets work with other immune cells, what their underlying molecular mechanisms are, and how to devise novel therapeutics to treat adenomyosis more effectively are unresolved questions that warrant future research.

Regardless, the ReTIAR notion and the roles of platelets in adenomyosis, as elaborated above, not only help us to piece together many seemingly unrelated findings, but also help us to see things that we were not aware of before. As demonstrated in several studies, anti-platelet therapeutics appears to be promising in treating adenomyosis, although their real efficacy would await much research. Still, given the spectacular failure of clinical trials of non-hormonal drugs in treating endometriosis/adenomyosis [23,310], it may be time to re-appraise the strategy, For example, anti-angiogenesis and anti-inflammation are often advocated, but the normal physiology of endometrium also requires angiogenesis and inflammation. Hence, a direct assault on angiogenesis and inflammation is likely to disrupt normal angiogenesis and inflammation needed for endometrial repair and, as such, cause collateral damage. On the other hand, as platelets are situated at the crossroads of proinflammatory and the resolution pathways during inflammation [311], the anti-platelet or anti-coagulation approach appears to more rational and beneficial.

## Data Availability

Not applicable.
